# A Case Report of Hemifacial Spasm Caused by Vestibular Schwannoma and Literature Review

**DOI:** 10.3390/brainsci12101347

**Published:** 2022-10-05

**Authors:** Xiaomin Cai, Yinda Tang, Hua Zhao, Zheng Chen, Haopeng Wang, Wanchun Zhu, Shiting Li

**Affiliations:** Department of Neurosurgery, Xinhua Hospital Affiliated to Shanghai Jiaotong University School of Medicine, The Cranial Nerve Disease Center of Shanghai Jiaotong University, Shanghai 200092, China

**Keywords:** hemifacial spasm, decompression, schwannoma

## Abstract

Background: Most cases of hemifacial spasm result from mechanical compression at the root exit zone of the facial nerve by vascular loops, and only a few cases are caused by vestibular schwannoma. Case presentation: We report a case of symptomatic hemifacial spasm induced by a small vestibular schwannoma that was totally resected. A 64-year-old man was admitted to our department with a 14-month history of symptomatic right-sided hemifacial spasm. During the process of microvascular decompression, no definite vessel was found to compress the facial nerve. By further exploration of regions other than root exit zone, a small vestibular schwannoma compressing the internal auditory canal portion of facial nerve from the ventral side was discovered. Resection of the tumor was then conducted. The symptoms of hemifacial spasm disappeared immediately after surgery. Conclusions: We should be aware that magnetic resonance imaging is not always precise and perhaps misses some miniature lesions due to present image technique limitations. A small vestibular schwannoma might be the reason for HFS, although preoperative magnetic resonance tomography angiography showed possible vascular compression at the facial nerve root. More importantly, a full-length exploration of the facial nerve is in urgent need to find potential compression while performing microvascular decompression for HFS patients.

## 1. Introduction

Hemifacial spasm (HFS) is one of the most common hyperactive cranial rhizopathies, typically characterized by unilateral, intermittent, involuntary twitching of the muscles innervated by the facial nerve [1,2]. At present, most cases of HFS result from mechanical compression at the root exit zone (REZ) of the facial nerve by vascular loops [3]. There are also a few reports about rare causes of secondary HFS, including cerebellopontine angle (CPA) tumor [4], glioma in the brain stem, etc. [5,6,7]. Based on previous studies, meningioma [8,9], lipoma [10], vestibular schwannoma (VS) [11], and epidermoid tumor [12] are common reasons for CPA tumor-induced HFS. However, the incidence of tumor-associated HFS is very low and cases of VS-related HFS are especially rare. In this paper, we report a case of HFS coexisted with a small VS, which was not found by preoperative magnetic resonance tomography angiography (MRTA), and which led to the occurrence of HFS. In addition, we also systematically reviewed the cases of VS-related HFS and further discussed the etiology and pathogenesis of this kind of neurological disorder.

## 2. Case Report

**Figure 1:**
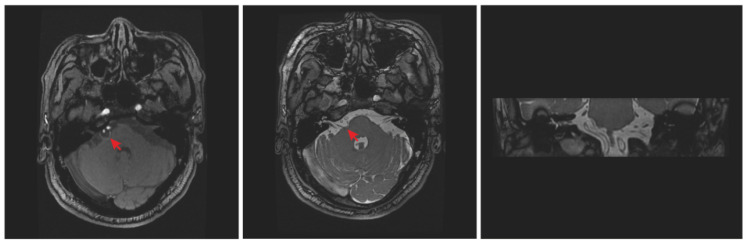
Preoperative MRTA showed that a cluster of cerebellar arteries compressed the root of the right facial nerve, but no sign of tumors was found (red arrowhead, left panel). The corresponding COSMIC sequence MRI at the same level was also provided (middle panel: axial section; right panel: coronal section).

This 64-year-old man was admitted to our department with a 14-month history of complete right-sided HFS. He was bothered by progressive intermittent twitching of the muscles on the right side of his face, which initially involved the right eyelid and gradually spread to the inferior part of the face ipsilaterally. At the early stage of the onset, the symptoms partially relieved after he received treatments of carbamazepine. However, spasm of the face muscles still relapsed after 3 months despite markedly increased drug doses. In addition, he also received intramuscular injections with botulinum toxin type A (BtA) once without obvious efficacy. On admission, we observed intermittent contractions of the muscles on the right side of the face and further neurological examination revealed no aberrant neurological signs. The typical symptoms, complemented with a definite abnormal muscle response (AMR) wave through preoperative electrophysiological monitoring, suggested that this patient should be diagnosed with typical HFS. Preoperative MRTA showed that a cerebellar artery passed through the REZ of the facial nerve and no obvious space-occupying lesions were found (Figure 1).

**Figure 2:**
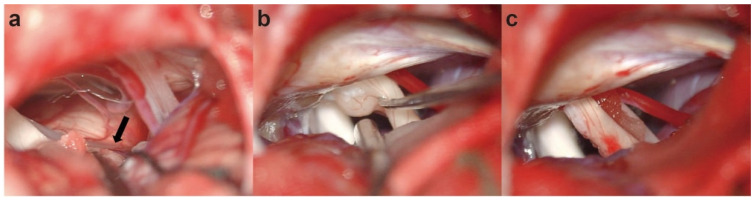
Intraoperative pictures: (**a**) no vascular compression was found in the root exit zone of the facial nerve (black arrowhead); (**b**,**c**) exploration and complete resection of the vestibular schwannoma.

A standardized right retrosigmoid approach was adopted, and the REZ of the facial nerve was routinely exposed using a microneurosurgical technique. Intraoperatively, we did not find blood vessel compression at the REZ of the facial nerve (Figure 2a). This is not in accordance with preoperative images. Upon further exposure, an unexpected small lesion was discovered with an ash grey mass, which was located between the facial-vestibulocochlear nerve complex and was close to the internal auditory canal (IAC). The facial nerve was compressed and displaced to the ventral side by this small lesion (Figure 2b). Considering that this lesion may be the cause of HFS, we decided to intraoperatively resect it after negotiation with the patients’ relatives. The lesion was then totally excised and the facial-vestibulocochlear nerves were well-preserved (Figure 2c). The intraoperative electrophysiological monitoring showed that the AMR waveform suddenly disappeared. No other compression sites were found after careful checking and closure was routinely performed.

**Figure 3:**
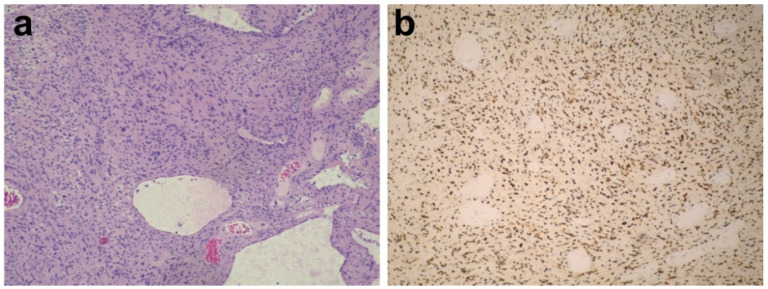
Histological features: (**a**) Hematoxylin and eosin (H&E) staining (original magnification × 100) showing the histological appearance of vestibular schwannoma; (**b**) immunohistochemistry showing positive reactivity for SRY-box transcription factor 10 (SOX10) (original magnification × 100).

The postoperative process was uneventful and the right HFS absolutely disappeared without any neurological deficits. Histopathologically, the lesion was verified to be a VS, which expressed verocay bodies and SOX10 proteins (Figure 3). Six months after operation, this patient presented no recurrence of HFS.

## 3. Discussion 

HFS is a common neurological disorder mostly caused by vascular compression, which often occurs at the facial nerve REZ of the pontomedullary junction [13,14]. Rarely, HFS can be induced by factors other than vascular compression itself, such as aneurysms, cysts, or space-occupying lesions in the CPA [3,6,10,15]. Among HFS caused by space-occupying lesions in the CPA, cases of VS are extremely rare. In this paper, we report a case of HFS induced by a small VS, whose symptoms were completely relieved after tumor removal. 

So far, cases of HFS in patients with VSs are rarely reported. By searching MEDLINE/PUBMED from 1985 to 2022, we retrieved studies that report, in total, 43 cases of HFS caused by or coexisted with VS (Table 1). Only T Nishi et al. [16] and Liu et al. [17] reported HFS underlying the mechanism of distorted brain stem by a huge VS compressing the REZ of the facial nerve. In this case, the brain stem acted as a medium, conducting force added by a contralateral tumor to the ipsilateral facial nerve root. VS was the only causative factor of HFS without vascular compression involved among the 24 cases. In the 43 cases with VS, the MVD procedure was performed in 12 cases in addition to tumor resection, and 14 cases without vascular compression at the REZ of the facial nerve underwent tumor excision only. Notably, gamma knife radiosurgery was used as an effective treatment to eliminate or debulk the tumor in nine cases. Complete relief of symptoms were acquired in 31 patients. However, two patients presented facial spasm again postoperatively after short-term relief of symptoms and no improvement was observed in two patients who were only treated with gamma knife radiosurgery. Lee et al. [4] thought that insufficient decompression of offending vessels for the purpose of preserving the perforating arteries and deposit of an unusual arachnoid thickening caused by inflammatory changes were responsible for HFS recurrence. According to our experience, associated vessel loops could be identified through careful exploration of the full length of the facial nerve in order to reduce the possibility of HFS recurrence. The symptoms of HFS patients with VS partially resolved in two cases after surgery. We cannot exclude the possibility that offending vessels were not discovered and hence the MVD procedure was not performed in these two patients. Undoubtedly, a satisfactory outcome was hard to achieve when the culprit was not discovered during the process of tumor resection.

In our patient, only MRTA and COSMIC sequence magnetic resonance imaging (MRI) were not sufficient for the identification of small occupying lesions at the CPA or IAC. Thus, we strongly recommend the additional use of high-resolution IAC MRI for HFS patients in order to preclude such lesions. The chance of wrong diagnosis would further decline when supplementary high-resolution IAC CT is also performed. In this article, we summarized 43 HFS cases caused by or coexisted with VS, shown in Table 1. Among them, VSs were clearly identified in 12 cases before MVD surgery. Other cases who received tumor resection or radiosurgery were also definitely diagnosed preoperatively. The corresponding results are included in Table 1. In our experience, the situation is really rare that VSs were found during MVD that were not picked up on preoperative imaging. We speculate that several potential reasons are responsible for this issue: (1) the volume of the occupying lesion was extremely small, so MRI could not identify it; (2) the patient has no common symptoms of VS, such as hearing loss, gait imbalance, etc.; or (3) high-resolution IAC CT or MRI was not preoperatively prescribed. Taken together, we recommend the usage of high-resolution IAC CT, MRI, and even contrast-enhanced MRI for HFS patients. The exploration of the full length of the facial nerve during MVD surgery could help identify the missed lesion.

The common complications for MVD are hearing loss, peripheral facial paralysis, cerebrospinal fluid leaks, lower cranial nerve dysfunction, and strokes, with an incidence of 1.9–20% [37,38,39], 3.63% [37,39], 2.5–10% [38], 0.5–1% [38], and 0–2.1% [38], respectively. No complication was noted in our case. Since 1 June 2009, we started to adopt the method of checking the whole course of the facial nerve while performing MVD for each HFS case [40]. This method helped us to identify the neurovascular conflict site located in regions other than the REZ [40,41]. Undoubtedly, the entire course of the facial nerve exploration technique required more dissection, and those delicate structures, such as the facial-vestibulocochlear nerve complex and caudal cranial nerves, brainstem, and cerebellum, as well as arteries, are more involved, which may take more operating time and risk [40]. Nevertheless, our previous study results demonstrated that the postoperative complications were not statistically different between the entire course exploration group and the pure REZ exploration group [40]. The incidence of postoperative complications depended more on the surgeon’s skill rather than manipulation alone [40,42]. With the increasing number of MVDs, the surgeon became more sophisticated and the total incidence of complications decreased accordingly [40,42]. In addition, exploration of the entire course of the facial nerve could also aid the discovery of miniature occupying lesions that were not found on preoperative imaging, just like the situation in this case. Notably, compared to the secondary HFS caused by VS, complication rates, such as the incidence of facial palsy in the primary HFS, were significantly lower according to the previous literature [30,43,44]. We speculate that the reasons are as follows: (1) The pathogenesis of secondary HFS is still controversial. Many authors believe that it is the vascular compression under the tumor that causes HFS [15] while other experts hold views that tumors may compress or distort either the REZ of the facial nerve or the facial nucleus, initiating HFS [45]. However, no matter which view is correct, the situation is quite different from the pure vascular compression in primary HFS. (2) It is often essential to debulk the tumor before probing and decompressing the facial nerve during the procedure of secondary HFS, which markedly increases the probability of involvement and injury of brain tissues and cranial nerves around the tumor.

In this case, although neurovascular conflicts were not initially found during operation, the outcome was fortunately adequate. The culprit appeared after careful exploration of the whole length of the facial nerve, which was an ash grey mass localized between the facial-vestibulocochlear nerve complex. After total resection of the lesion, the spasm disappeared immediately without any neurological deficits. The histopathological results that returned are suggestive of a VS. 

## 4. Conclusions

We reported a rare HFS case due to compression of facial nerve caused by VS. The key to successful treatment of HFS was as follows: (1) a full-length exploration of the facial nerve so as to avoid missing compression sites other than the REZ of facial nerve; (2) consideration of the cause of VS, especially in those patients whose preoperative image did not show signs of an occupying lesion and those with no definite vascular compression found during MVDs.

## Figures and Tables

**Table 1:** Summary of reports of hemifacial spasm caused by or coexisted with vestibular schwannoma.

Year	Author	Sex/Age	Feature of VS	Compression of FN	Management	Outcome
1987	T Nishi [16]	/	Contralateral VS	VS	Removal of the VS	Cure after 14 days
1987	A Morita [18]	/	Ipsilateral VS	VS	Removal of the VS	/
1988	Y. Sugiura [19]	Female/63	Ipsilateral VS	VS	Removal of the VS	Immediately cure
1988	Y. Sugiura	Female/53	Ipsilateral VS	VS	Removal of the VS + HFA	Immediately cure
1992	Shinji Nagata [20]	Female/29	Ipsilateral VS	VS	Removal of the VS	Immediately cure
1995	M. Samii [21]	Female/54	Ipsilateral VS	Arterial branches of AICA	Removal of the VS + MVD	Immediately cure
1995	M. Samii	Female/58	Ipsilateral VS	Arterial branches of AICA	Removal of the VS + MVD	Immediately cure
1995	M. Samii	Male/42	Ipsilateral VS	Arterial branches of AICA	Removal of the VS + MVD	Immediately cure
1995	M. Samii	Female/47	Ipsilateral VS	Arterial branches of AICA	Removal of the VS + MVD	Immediately cure
2001	Néstor Gálvez-Jiménez [11]	Female/67	Ipsilateral VS	VS	Gamma Knife Radiosurgery + botulinum toxin type A	/
2004	S. Peker [22]	Male/49	Ipsilateral VS	VS	Gamma Knife Radiosurgery	Delayed cure
2006	Bruce E Pollock [23]	Male/66	Ipsilateral VS	VS	Radiosurgery + Removal of the VS	Delayed cure
2006	Bruce E Pollock	/	/	VS	Radiosurgery	Recurred
2006	Jonathan G. Bull [24]	Female/12	Ipsilateral VS	VS	Removal of the VS	Immediately cure
2009	In-Bo Han [25]	/	/	VS	Removal of the VS	Immediately cure
2010	Hongyan Han [26]	/	/	VS + branches of cerebellar arteries	Removal of the VS + MVD	/
2010	Hongyan Han	/	/	VS + branches of cerebellar arteries	Removal of the VS + MVD	/
2010	Seung Hwan Lee [4]	Female/60	Ipsilateral VS	VS + PICA	Removal of the VS + MVD	Immediately cure
2010	Seung Hwan Lee	Female/60	Ipsilateral VS	AICA + VA	Removal of the VS + MVD	Recurred
2012	Cheng-Siu Chang [27]	Female/51	Ipsilateral VS	VS	Gamma Knife Radiosurgery	Failing
2012	Cheng-Siu Chang	Male/50	Ipsilateral VS	VS	Gamma Knife Radiosurgery	Delayed cure
2013	F. A. Zeiler [28]	Female/52	Ipsilateral VS	VS	Gamma Knife Radiosurgery	Worsening
2013	Pierre Bouchetemble’ [29]	/	Ipsilateral VS	VS	Removal of the VS	/
2015	Frederick A. Zeiler [30]	Female/56	Ipsilateral VS	VS + PICA	Removal of the VS + MVD	Delayed cure
2016	Constantin Tuleasca [31]	/	/	VS	Gamma Knife Radiosurgery	Partial relieving
2016	Ming-Xing Liu [32]	Female/44	Ipsilateral VS	VS + AICA	Removal of the VS + MVD	Immediately cure
2016	Ming-Xing Liu	Female/51	Ipsilateral VS	VS	Removal of the VS	Partial relieving
2016	Ming-Xing Liu	Male/58	Ipsilateral VS	VS	Removal of the VS	Partial relieving
2016	Ming-Xing Liu	Male/59	Ipsilateral VS	VS	Removal of the VS	Immediately cure
2018	Jiang Liu [17]	Female/58	Ipsilateral VS	AICA	Subtotal removal of the VS	Immediately cure
2018	Jiang Liu	Male/43	Ipsilateral VS	VS	Gross total removal of the VS	Immediately cure
2018	Jiang Liu	Female/39	Ipsilateral VS	AICA	Gross total removal of the VS	Immediately cure
2018	Jiang Liu	Female/68	Ipsilateral VS	AICA	Subtotal removal of the VS	Immediately cure
2018	Jiang Liu	Male/53	Ipsilateral VS	PICA	Gross total removal of the VS	Immediately cure
2018	Jiang Liu	Male/71	Ipsilateral VS	VS	Gross total removal of the VS	Immediately cure
2018	Jiang Liu	Female/49	Ipsilateral VS	AICA	Gross total removal of the VS	Immediately cure
2018	Jiang Liu	Female/60	Ipsilateral VS	PICA	Subtotal removal of the VS	Immediately cure
2018	Jiang Liu	Female/57	Ipsilateral VS	AICA	Gross total removal of the VS	Immediately cure
2018	Jiang Liu	Male/62	Contralateral VS	VS	Gross total removal of the VS	Delayed cure
2018	Cheng-Wei Huang [33]	/	/	VS	Gamma Knife Radiosurgery	Cure after 3 years
2021	Carlos Candanedo [34]	Female/27	Ipsilateral VS	VS + AICA	Removal of the VS + MVD	Immediately cure
2022	Khairunnisak Misron [35]	Male/68	Ipsilateral VS	VS + AICA	Removal of the VS + MVD	Immediately cure
2022	Roser, Florian [36]	Female/42	Ipsilateral VS	VS	Removal of the VS	Immediately cure

AICA: anterior inferior cerebellar artery; FN: facial nerve; HFA: hypoglossal-facial nerve anastomosis; MVD: microvascular decompression; PICA: posterior inferior cerebellar artery; VA: vertebral artery.

## Data Availability

The data presented in this study are available upon request from the corresponding author.
